# How should the health system react to informal drug dispensaries? The case of self-medication in Yogyakarta Province

**DOI:** 10.1186/1471-2458-12-S2-A16

**Published:** 2012-11-27

**Authors:** Eunice Setiawan, Mubasysyir Hasanbasri, Laksono Trisnantoro

**Affiliations:** 1 Center for Health Service Management, Faculty Medicine, Universitas Gadjah Mada, Jogjakarta, Indonesia; 2 Health Policy and Management, Faculty of Medicine, Universitas Gadjah Mada, Jogjakarta, Indonesia

## Background

The government seeks to build a healthy community through quality, generic drugs that are available, accessible and affordable. On the other hand, self-medication among different community segments has grown in popularity. In this paper, we want to argue that if the government fail to regulate and to supervise informal drug dispensaries, the community - particularly the poor - is more likely to gain harmful effects through the practice of self-medication and the use of unregulated informal drug stores. This study aims to: determine the pattern of self-medication in the province of Yogyakarta, identify the type of drug dispensaries that self-medication users seek in their communities, and to evaluate the effectiveness of ministry of health’s regulations and supervisions on drug dispensaries.

## Materials and method

This study used data from the 2009 Indonesian Family Life Survey available from the Rand Corporation. Qualitative data were obtained from field observations and in-depth interviews with informal drug dispensaries and officers from provincial and district health authorities.

## Results

The practice of self-medication is popular among urban and suburban population. The prevalence of self-medication varies across districts. The sub-urban districts shows a relatively high use of self-medication (Kulonprogo, 60%; Gunung Kidul (69%); Bantul (65%); and Sleman (69%). It is surprisingly that the use of self-medication practice is 90% in Yogyakarta city. An increasing number of informal drug dispensaries run without necessary supervision and regulation both from district and provincial health authority. Community dispensaries were found to obtain drug supply from non-authorized pharmaceutical distributors.

## Conclusions

There is an increasing tendency for the community, the poor and the better off, to use self-medication as health seeking practice for minor illness. In contrast, the Ministry of Health lacks control over informal drug dispensaries. It is recommended that the government puts in place regulation and supervision strategies toward community based drug dispensaries.

